# Cohort Differences in Daily Life: Older Adults Have Higher Affect Variability Than 18 Years Ago

**DOI:** 10.1093/geroni/igab046.3672

**Published:** 2021-12-17

**Authors:** Johanna Drewelies, Rachel Koffer

**Affiliations:** 1 Humboldt University of Berlin, Humboldt University Berlin, Berlin, Germany; 2 Arizona State University, Phoenix, Arizona, United States

## Abstract

Lifespan psychological and life course sociological perspectives have long acknowledged that individual functioning is shaped by historical and socio-cultural contexts. Secular increases favoring later-born cohorts are widely documented for general well-being (among older adults). However, little is known about secular trends in short-term fluctuations in daily affective well-being and whether historical changes have occurred in young, middle-aged, and older adults alike. To examine these questions, we compared data from two independent national samples of the NSDE survey obtained 18 years apart (1995/96 vs. 2013/14) and identified case-matched cohorts (per cohort, n = 782, aged = 23–75 years) based on age and gender. We additionally examine the role of economic and health resources for cohort differences in affective variability. Results revealed that later-born cohorts report higher variability in daily negative affect than did those 18 years ago. In contrast, no cohort differences emerged in variability in daily positive affect. We conclude from our national US sample that secular trends in affect variability do not generalize unanimously to different timescales across adulthood. We discuss possible underlying mechanisms and practical implications.

